# Automatic Detection and Reproduction of Natural Head Position in Stereo-Photogrammetry

**DOI:** 10.1371/journal.pone.0130877

**Published:** 2015-06-30

**Authors:** Tai-Chiu Hsung, John Lo, Tik-Shun Li, Lim-Kwong Cheung

**Affiliations:** Discipline of Oral and Maxillofacial Surgery, Faculty of Dentistry, The University of Hong Kong, Pokfulam, Hong Kong; FIOCRUZ, BRAZIL

## Abstract

The aim of this study was to develop an automatic orientation calibration and reproduction method for recording the natural head position (NHP) in stereo-photogrammetry (SP). A board was used as the physical reference carrier for true verticals and NHP alignment mirror orientation. Orientation axes were detected and saved from the digital mesh model of the board. They were used for correcting the pitch, roll and yaw angles of the subsequent captures of patients’ facial surfaces, which were obtained without any markings or sensors attached onto the patient. We tested the proposed method on two commercial active (3dMD) and passive (DI3D) SP devices. The reliability of the pitch, roll and yaw for the board placement were within ±0.039904°, ±0.081623°, and ±0.062320°; where standard deviations were 0.020234°, 0.045645° and 0.027211° respectively. **Conclusion**: Orientation-calibrated stereo-photogrammetry is the most accurate method (angulation deviation within ±0.1°) reported for complete NHP recording with insignificant clinical error.

## Introduction

Recently, a new method for recording Natural head position (NHP) [1–10] in stereo-photogrammetry (SP) was suggested [11]. The idea was to use the physical references of a pre-captured digital model of a board to correct the orientation of subsequent facial surface models. The board was placed in a way that it was vertically placed and parallel to a hanging mirror at the center of the SP devices. The mirror was an external reference for patients to reproduce their own NHP by looking straight into the mirror at their own eyes in a balanced position [12]. When the patients reproduced their own NHP, this method was able to record this posture accurately. The achieved overall accuracy of physical references derived from the board [11], to the axes defined by gravity and mirror orientations, was within ±0.2°; where standard deviation was around 0.11°. These were considered to be clinically insignificant. The stereophotogrammetric NHP method possesses many unique advantages over traditional approaches: 1) radiation-free; 2) patients can be in their own NHP for capturing of facial surface models without the need for any markers/sensors; 3) only one reference board recording is needed after camera calibration [13–14]. We can further use the obtained orientation as references for other imaging modalities such as CT scans in surgical planning (e.g. 3dMDvultus, 3dMD, USA). Although high accuracy can be achieved with this approach, there are some manual steps in correcting the orientation of patients’ facial surfaces. To simplify the procedure in order to minimize operator variability, we developed an automatic orientation calibration method in this paper. The method consists of two components: 1) a specially designed reference board; and 2) a computation method in physical references detection and patients’ facial surfaces correction. The aim of this paper was to report this technique and its reliability.

## Material and Method

**Fig 1:**
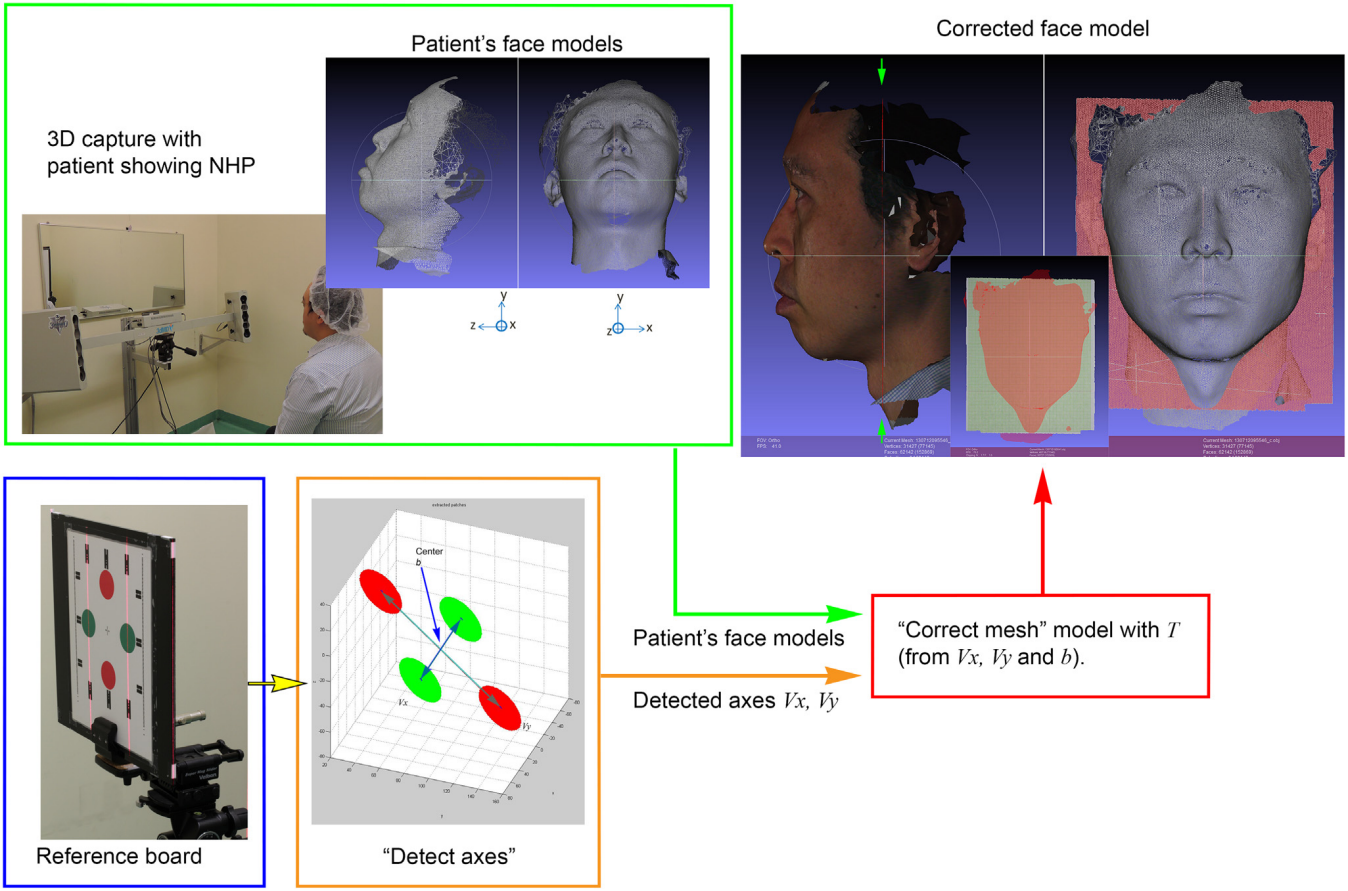
The workflow of the proposed automatic detection and reproduction of NHP. Lower left: The digital mesh model of the reference board was recorded. True horizontal ***v***_*x*_ and vertical ***v***_*y*_ axes were detected and saved. Then, patients’ face models (upper left) were corrected (right) according to the transformation **T**.

The individuals (appears in Fig 1) in this manuscript have given written informed consent to publish these case details. In fact, they are the first two authors of the manuscript. Consent forms for publication are also signed and keep securely. In this study, the 3D surface scans were performed using 3dMDface (3dMD USA) and DI3D (Dimensional Imaging, UK). The former one is an active SP device [15, 16] which projects infrared pseudo random patterns onto the subjects for 3D reconstruction. The latter one is a passive SP device [17] which does not project patterns but requires that the surfaces contain sufficient texture contrast in order to work. For these SP devices, different methods were used for their geometric camera calibration. Orientation of the captured surfaces usually followed the calibration targets’ orientation which was manually placed, without aligning to any physical references (e.g. true vertical). For 3dMD, the reconstructions were tilted backwards approximately 45°. For DI3D, the reconstructions appeared to be in the correct orientation, but were found to be 1°–4° deviated from all reference axes. These orientation biases remained constant until the next geometric camera calibration. Due to the manual placement of the geometric calibration targets, the orientation biases for each calibration were usually different.

In the proposed method, the materials and tools are listed as follow:

1. SP devices (3dMD and DI3D),
2. Two/three 360° 3-plane leveling and alignment lasers (Bosch GLL3-80P, Germany),
3. NHP alignment mirror placed at the center of the 3D camera,
4. High quality acrylic board with alignment patterns,
5. Tripod equipped with a 3D geared head (Manfrotto 410 junior geared head), two-way focusing adjuster (Velbon Super Mag Slider) and vice clamp,
6. Simulation programs (Mathworks Matlab R2012b, USA) for orientation detection and corrections.

### A. Reference board alignment

**Fig 2:**
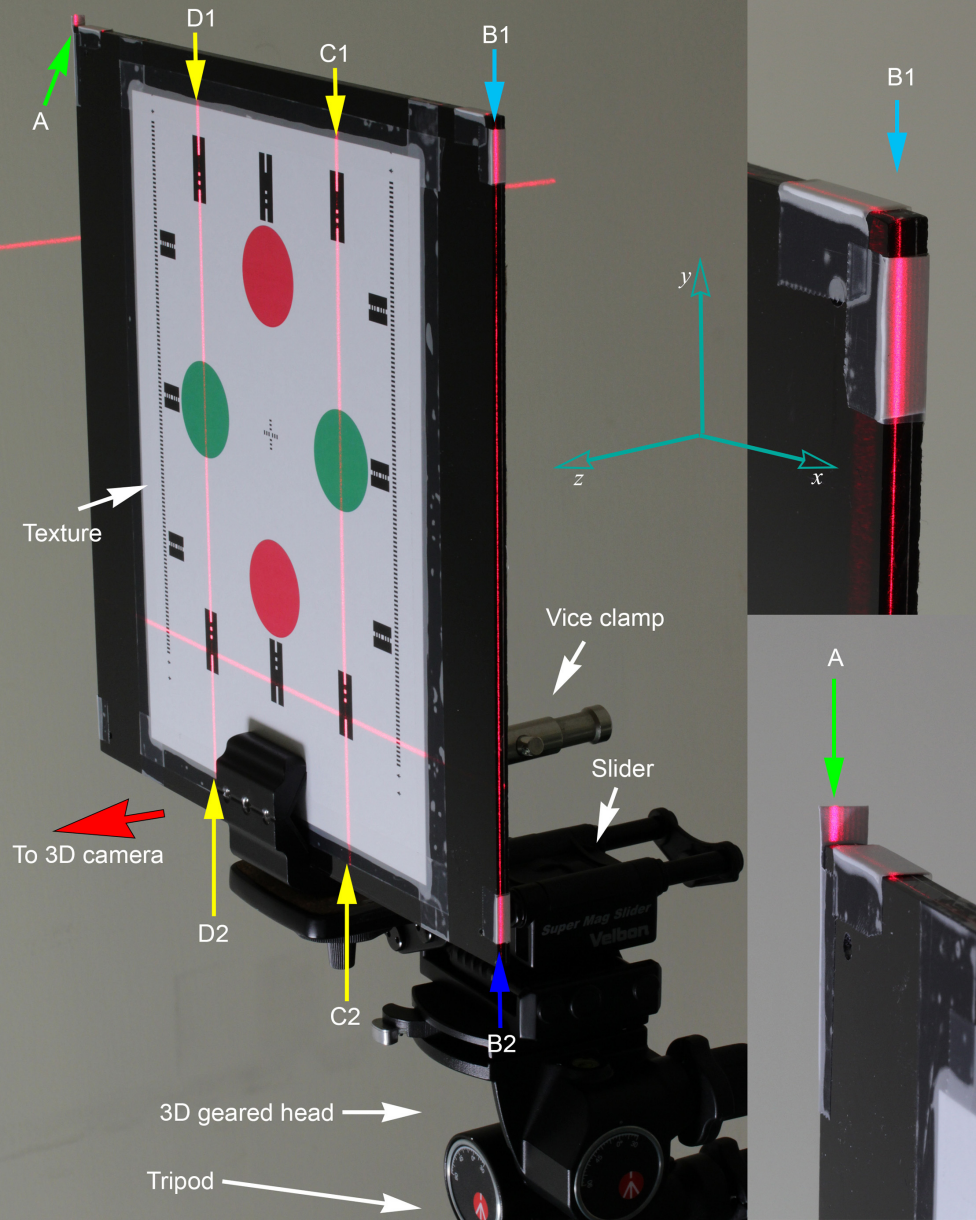
Set up of the reference board. The reference board was taped with color patterns on the front side facing the SP device cameras. Side grooves were crafted by using surface gauge at the side and top edge. Short paper tags were taped near the edges for angulation matching with laser beams. It was held by a vice clamp, connected to a two-way focusing slider for fine horizontal positional adjustments; and 3D geared head for angulation adjustments. Laser beams A (bottom right) and B1–B2 were projections from V2 which was aligned to be parallel to the mirror plane. Laser beam C1–C2 and D1–D2 were projections from front alignment lasers. Yaw angle was aligned with A-B1 (right) and top board edge; pitch angle was aligned with B1–B2 and side groove; roll angle was aligned with C1–C2, D1–D2 and dotted lines on card paper.

To enable human subjects to be in their own NHP, a mirror was installed at the center of the SP device. The subjects were in their NHP when looking straight ahead at their own eyes through the mirror [1–11]. Physical references, i.e. the true verticals and mirror plane heading, were needed to fully characterize subjects’ posture. They were aligned with a customized reference board which was then held with a vice clamp and was in turn mounted on a tripod equipped with a 3D geared head and a two-way focusing adjuster (Fig 2). The 3D geared head and the adjuster were used to fine tune the angulation and horizontal positioning respectively. A card paper was printed (HP LaserJet 100 Color MFP M175a, A4 180gsm plain paper) with colored patterns of two red and green discs (Fig 2) along the vertical and horizontal axes respectively. Dotted lines along the axes were used as guides for alignment with laser beams. The card paper was designed using Adobe Photoshop CS6 in 300dpi. The dimension parameters of the discs were: 450px (1.5”) in diameter; center-to-center distances of red discs and green discs were 1430px (4.76”) and 1200px (4”) respectively; 9 vertical laser alignment marks: 20x10mm. This dimension was selected so that the discs would span the whole field of view of the SP devices.

**Fig 3:**
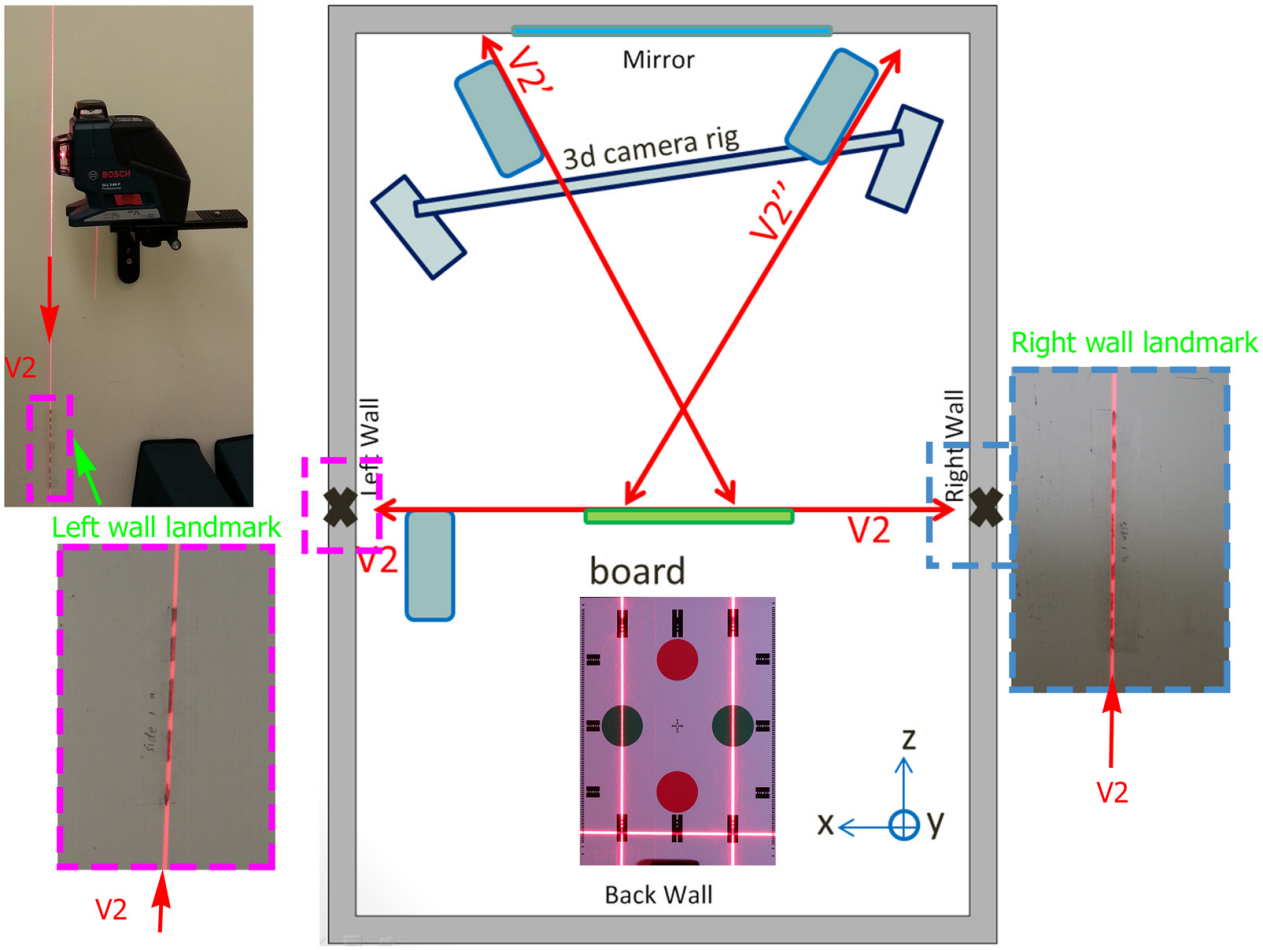
Top view of 3D camera room. The reference board was placed at the capturing position which was aligned with laser beams. The vertical laser plane V2 from side laser was aligned with side wall landmarks (dotted lines marked with pencil) which indicate the mirror plane (pitch and yaw). Another vertical laser plane V2’ and V2” from front lasers indicate true verticals on the reference board pattern (roll). Two front alignment lasers offer cross validation of the angulation adjustments.

**Fig 4:**
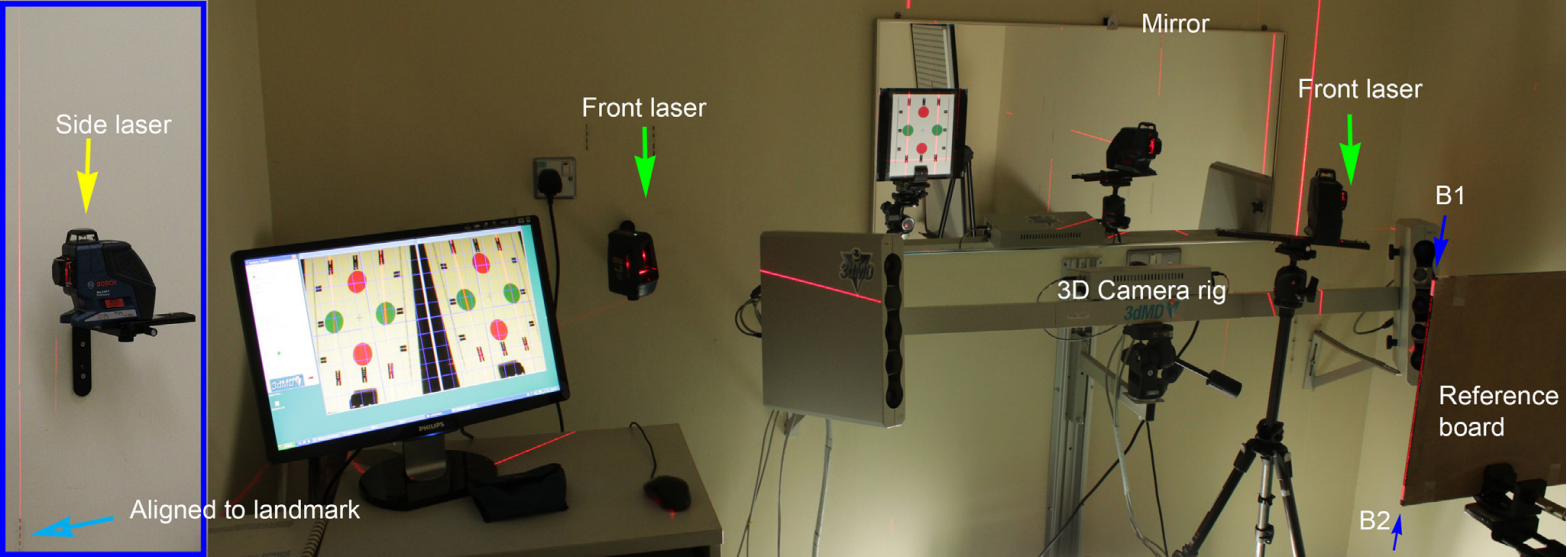
Setting of 3D camera room. The side laser was elevated to a higher position than the reference board such that the laser plane V2 can beam up the side (pitch) and top edge (yaw) of the board. It was aligned to the side wall landmarks such that V2 was parallel to the NHP mirror plane. Another alignment lasers were placed next to the 3D camera rig for projecting laser beam indicating true verticals for roll angle.

In order to transfer the physical references to the board, it was required that the indication of true verticals and mirror heading were available at the capture position. For the true verticals, the alignment lasers project vertical planes automatically. For the mirror heading, we adopted the same method as described in the original SP NHP recording method [11] for placing landmarks in the 3D capturing room in relating the NHP alignment mirror plane to the capturing position (position of board in Fig 3). The alignment laser (Bosch GLL3-80P, Germany) was first placed in the capturing position. It was adjusted in such a way (Fig 4 in [11]) that when the laser was turned on, the laser line on the back wall directly from the vertical laser plane (V1) would overlap with the reflected laser plane V1 from the mirror. This vertical laser plane was therefore perpendicular to the plane of the mirror. The position of the second vertical laser plane V2, which was engineered to be perpendicular to the first laser plane V1, was then marked onto the side walls. These landmarks were marked onto the side walls with a pencil as dotted lines (magenta and blue boxes in Fig 3) such that any laser beam deviations could easily be identified at their projection edges. Hence, the mirror plane was transferred to the capturing position with the indications of side wall landmarks. The alignment laser was then moved to the left (or right) side in order to make space for 3D capture of the board (Fig 3). It was also aligned with the side wall landmarks and elevated to a position such that the laser was able to light up the top and side of the board edges as shown in Fig 4.

Moreover, two additional alignment lasers were placed next to the 3D camera rig for indicating true verticals (V2’, V2”) on the reference board pattern. The position and angulation of the board were adjusted to match with the laser beams. Laser beams A-B1 and B1–B2 (Fig 2) were projections from V2 (Fig 3), which was aligned to be parallel to the mirror plane. Laser beam C1–C2 and D1–D2 were projections from the front alignment lasers.

The reference board angulation was aligned with the laser beams using the following procedure (Fig 2),

### Procedure 1

1. Align yaw angle with A-B1 and top board edge.
2. Align pitch angle with B1–B2 and side edge. It was also cross validated with the agreement of the front alignment lasers and the patterns.
3. Align roll angle with C1–C2, D1–D2 and dotted lines on the card stock.

After alignment, 3D capture of the reference board was performed with the lasers turned off. The captured digital mesh model of the board was saved in Wavefront (OBJ) format for subsequent calculations. The board was then removed to make space for subsequent facial captures.

### B. Digital processing on orientation detection and correction

After the device geometric camera calibration, the aligned reference board was scanned with the SP device. The digital processing on the captured digital mesh model consisted of two parts: 1) Physical reference detection; and 2) orientation correction. In the first part, axes corresponding to the physical references were detected from the reference board digital mesh models and saved. In the second part, the saved parameters were used to correct subsequent patients’ facial models. We implemented these two processing steps in Matlab (Mathworks, USA).

#### (i) Physical reference axes detection

**Fig 5:**
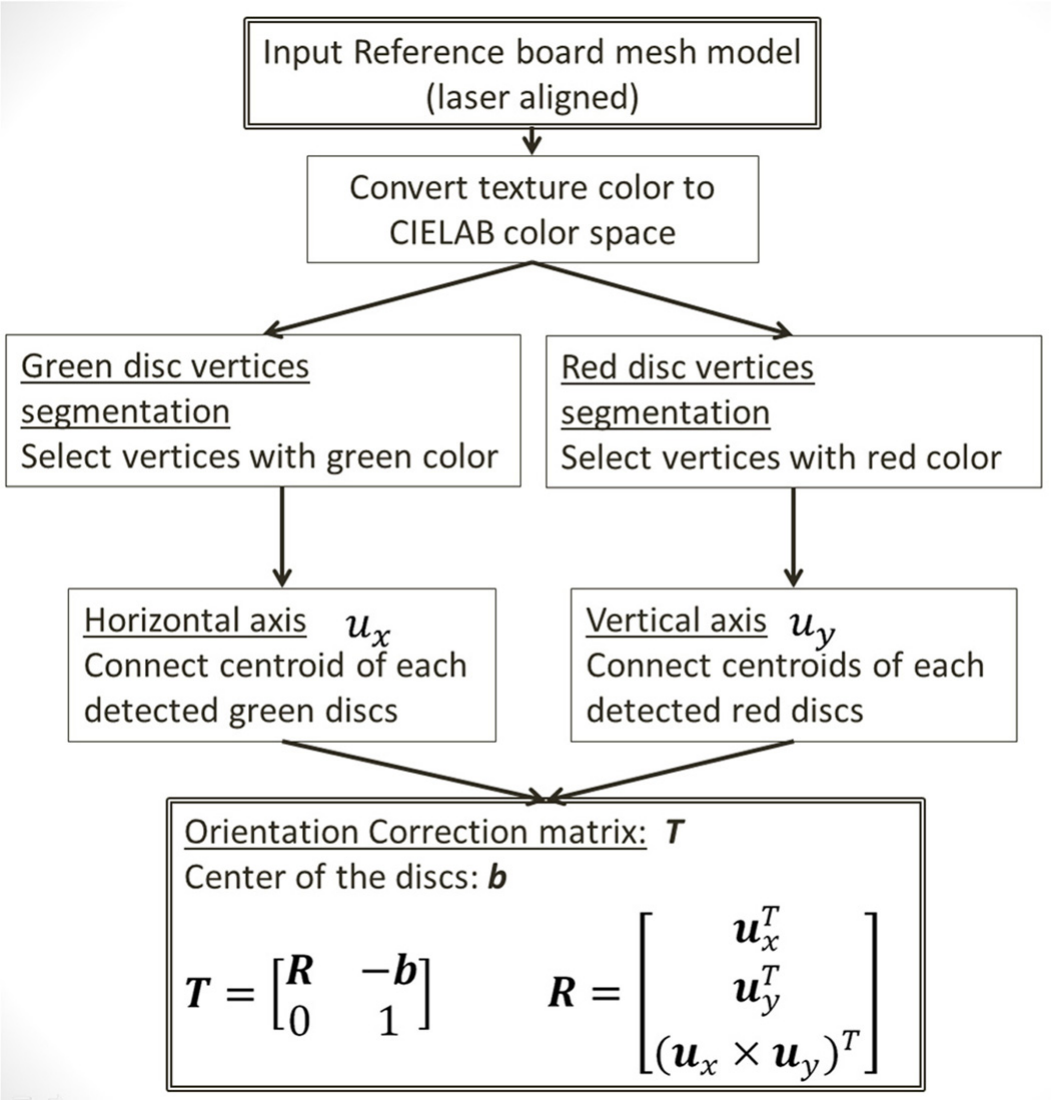
Flowchart of computing the orientation correction matrix *T* from laser aligned reference board.

Following procedure 1, the red discs were aligned along with the true verticals (roll and pitch), whereas green discs were aligned with the true horizontal (roll and yaw). Moreover, the discs were on a vertical plane (yaw) which was parallel to the mirror. This is because the board has been aligned with the laser plane V2 (Fig 3). The 3D vectors, ***v***_*x*_ and ***v***_*y*_, connected through the centroids of each group of the discs indicated the true horizontal and vertical axes. As the SP devices do not consider physical orientation, these axes were tilted (Fig 1, upper left) in the 3D reconstructed surfaces. To extract these vectors (Fig 5), the associated texture colors of the 3D surfaces were first converted into CIELAB color space [18]. 3D vertices of the green and red discs were segmented according to the chromaticity components a* and b* of the associated texture color. In our configuration, the angle of chromaticity vector (a*, b*) of red and green colors were distributed at 0.2 rad (±0.4 rad) and π-0.6 rad (±0.4 rad) respectively. For the adopted SP devices, lighting and exposures were under well-control. The captured images were very clean. Camera distortions and perspective effects were effectively compensated by the SP devices for enabling the 3D surface reconstruction. Therefore, discs centroids could reliably be calculated by simply taking the mean of the segmented 3D vertices.

#### (ii) Digital mesh model correction

Assuming that the SP devices were able to reconstruct the 3D surface with negligible distortion errors, it sufficed to adopt a rotation transformation for the orientation correction, which corrected the physical reference axes ***v***_*x*_ and ***v***_*y*_ back to the X and Y axes in 3D Euclidean space. That is, in homogenous coordinates,

$$T=\left[\begin{array}{cc} R & -b\\ 0 & 1 \end{array}\right]$$ (1)

where ***b*** is the center of the four discs; **0** is 1x3 matrix with all elements zero and **R** is the rotation matrix,

$$R=\left[\begin{array}{c} u_{x}^{T}\\ u_{y}^{T}\\ {\left(u_{x}^{}\times u_{y}^{}\right)}^{T} \end{array}\right]$$ (2)

where ***u***_*y*_ is the unit vector of ***v***_*y*_; and ***u***_*x*_ is obtained from Gram-Schmidt process on ***u***_*y*_ and ***v***_*x*_. Both unit vectors ***u***_*x*_ and ***u***_*y*_ were set to be positive in *x* and *y* components respectively. The correction of each vertices **v**: = (*v*_1_,*v*_2_,*v*_3_,1)^*T*^, of the reference board and patients’ digital mesh model were computed by,

w=Tv (3)

where we denote **w** as the corrected vertices. Since the SP devices were found to have high reproducibility from previous studies [13–14], this matrix is valid for the subsequent captured face models until next geometric camera calibration.

## Validation Analysis

### A. Accuracy analysis

The accuracy of the proposed method depended on several components: the accuracy of the alignment lasers, reliability of 3D surface reconstruction, reference board placement and computer orientation axes detection. For the adopted alignment lasers (Bosch GLL-3-80P), the accuracy was rated to be within: ±0.2mm/m (±0.011459°) from the true verticals/horizontal. They were calibrated in factory using devices with traceable accuracy. The angulation accuracy for each alignment steps with alignment lasers was read as ±0.011459° to the true verticals/horizontal. In the following experiments, the reference board was aligned with the laser beams projected on its marks. The pitch and roll angulations were directly aligned with the alignment laser (angulation accuracy ±0.011459°). The yaw angle was aligned to the side wall landmarks which were inferred from the NHP alignment mirror plane (angulation accuracy ±0.022918°). The overall accuracy of the proposed method was the sum of laser alignments and the reliability of the reference board placement and orientation axes detection. We used the following procedures to find out the reference axes detection reliability,

### Procedure 2

1. Side walls landmarks were set and the NHP alignment mirror were kept fixed.
2. The SP device was calibrated.
3. Reference board alignment.
4. Perform 3D scans for *M* times.
5. Repeat *N* trails from step 3.
6. Compute all orientation axes ***u***_*x*_ and ***u***_*y*_ with the simulation program.
7. Calculate angulation deviations of the axes in pitch, roll and yaw angles relative to the mean axes.

Assuming the alignment lasers were in good condition; mirrors, cameras and walls landmarks were fixed during the tests. Each laser beams and alignment patterns were matched appropriately. Patterns and the reference board were also checked that they were not significantly deformed due to handling and environment conditions. From the above procedure, we were able to characterize the overall accuracy of board placements in terms of orientations deviation. Components of the orientation axis ***u***_*y*_, were used to calculate the roll and pitch angles with $\theta_{roll}=\text{arctan}\left(u_{y,2}/u_{y,1}\right)$ and $\theta_{pitch}=\text{arctan}\left(u_{y,2}/u_{y,3}\right)$ respectively. The yaw angle was calculated with $\theta_{yaw}=\text{arctan}\left(u_{x,1}/u_{x,3}\right)$ where we denote the detected orientation axes as **u**_*x*_:=(*u*_*x*,1_,*u*_*x*,2_,*u*_*x*,3_)^*T*^ and **u**_*y*_:=(*u*_*y*,1_,*u*_*y*,2_,*u*_*y*,3_)^*T*^.

### B. Validation with dummy plastic head

**Fig 6:**
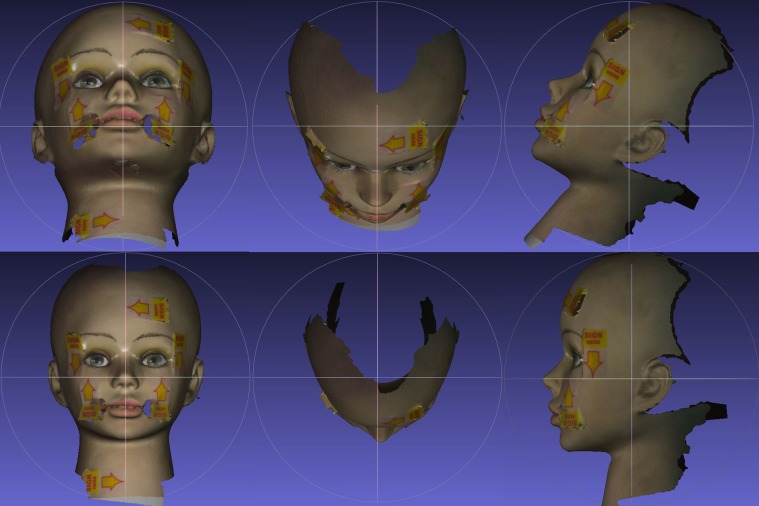
Validation of the automatic orientation method on a plastic head. The alignment laser was placed at the center of SP device (which was perpendicular to the NHP mirror) and were indicated with the arrow stickers. Top row: frontal, top and left lateral views of reconstructed facial surface from 3dMD. Bottom row: corrected surfaces with the proposed method.

Validation of the proposed method was illustrated in Fig 6. An alignment laser was placed at the center of the SP device with the laser beams (vertical and horizontal) aligned to be perpendicular to the mirror plane. The projected laser beams on the plastic head were indicated with arrow stickers. The captured plastic head surface was corrected using the detected axes. In the figure, we can see that the captured surfaces were corrected as they would appear in the physical world. The arrow stickers were pointing to the true horizontal and verticals.

### C. Axes detection reliability

Besides the plastic head validation, we investigated the axes detection reliability using the active and passive SP devices respectively. They were checked with multiple shots on the still aligned reference board with Procedure 2. The angular deviations in pitch, roll and yaw were found to be within ±0.01199°, ±0.16296°, ±0.007214° respectively; where standard deviations were 0.00433°, 0.07639° and 0.00287° respectively for 3dMD (M = 60). For DI3D (M = 30), the deviations in pitch, roll and yaw were within ±0.07393°, ±0.16327°, ±0.04361° respectively; and standard deviations were 0.02831°, 0.06542° and 0.02385°. The descriptive statistics of the experiments are shown in Table 1. Higher angulation deviation in roll was found. It is probably due to sampling error of disparity matching during surface reconstruction. The axes detection deviations can be reduced by averaging the detected axes from multiple shots. Averaging of five shots was found to be sufficient to reduce the roll angulation errors to similar level of pitch and yaw’s.

**Table 1:** Descriptive statistics of angulation deviations of the detected axes of still reference board for 3dMD (M = 60) and DI3D (M = 30).

**Angulation deviation for 3dMD**			**M = 60**	
	**SD/°**	**Median/°**	**Min/°**	**Max/°**
Pitch	0.004333	-0.000082	-0.011987	0.008351
Roll	0.076394	-0.005672	-0.162960	0.157693
Yaw	0.002873	-0.000082	-0.006768	0.007214
**Angulation deviation for DI3D**			**M = 30**	
	**SD/°**	**Median/°**	**Min/°**	**Max/°**
Pitch	0.028309	0.002814	-0.047836	0.073933
Roll	0.065417	0.003076	-0.163268	0.119684
Yaw	0.023846	-0.003870	-0.041924	0.043610

### D. Reproducibility

**Fig 7:**
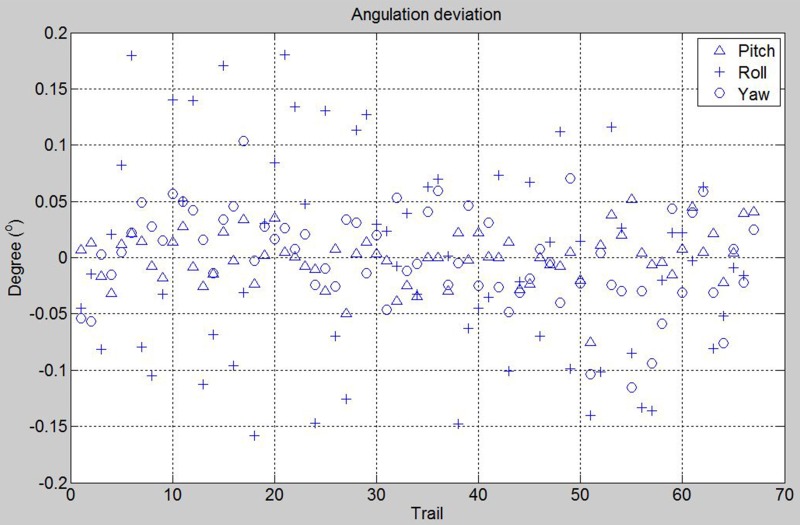
Reference board angulation deviations for using one front alignment laser (3dMD). 75 trails were performed by 3 investigators, 8 were excluded due to bad exposures and apparent misalignment.

The inter-operator reproducibility was tested among three operators (TCH, JL, TSL). We performed the reference board alignment for a total of *N* = 75 (*M* = 1) in 5 days. The number of trial for the operator 1, 2 and 3 are respectively 49, 9 and 9. Operator 1 performed 52 alignments on the first 2 days and the other operators performed 12 and 11 alignments respectively on the fifth day. The SP device was firstly calibrated on the first day. No more geometric camera calibration was done in this session. Eight of the captures were excluded due to bad exposures. Three re-alignments of laser to side walls landmarks were performed at the first, 38^th^ and 53^rd^ trials. Roll angle was aligned with **one** front alignment laser. Since the SP device possesses high reproducibility and only one geometric camera calibration was done at the beginning, we were able to see the reproducibility among operators whether contain significant bias in reference recording. In Fig 7, it shows the angulation deviations to the mean for the trials. The angulation deviation of board placement in pitch, roll and yaw were within ±0.075°, ±0.18°, ±0.12° respectively; and standard deviations were 0.02335°, 0.09239° and 0.04249° respectively. The descriptive statistics of the experiments are shown in Table 2. We can see that the reproducibility was very high even several side laser alignments were performed.

**Table 2:** Descriptive statistics of angulation deviations of reference board alignment for 3dMD, N = 67.

Angulation deviation for three operators			N = 67	M = 1
	SD/°	Median/°	Min/°	Max/°
Pitch	0.023345	0.001	-0.075296	0.051859
Roll	0.092389	-0.007924	-0.158411	0.179983
Yaw	0.042495	0.003002	-0.115625	0.10353

One sample t-test was performed on the results of each angulation deviation of pitch, roll and yaw from the three operators. The null hypothesis was that the mean angulation deviations were not different to 0°. From Table 3, the mean angulation deviation was not significantly different statistically to 0° with 95% CI, p > 0.05 except for operator 2 in yaw angle. Despite of the fact that significant difference was found for operator 2, the range of angulation deviation for all operators was still very small: i.e. ±0.12°. The largest mean differences were 0.014°±0.024° in pitch, 0.051°±0.090° in roll and 0.053°±0.042° in yaw.

**Table 3:** One sample t tests to determine whether the angulation deviation in pitch, roll and yaw from the three operators were different to 0°.

	Mean	SD	t	p-value	95% Confidence Interval of the Difference	
					Lower	Upper
**Pitch**						
Operator 1	-.00294015	.019512315	-1.055	.297	-.00854475	.00266444
Operator 2	.00207038	.036709703	.169	.870	-.02614719	.03028796
Operator 3	.01392989	.024248715	1.723	.123	-.00470932	.03256911
**Roll**						
Operator 1	.00681882	.094297369	.506	.615	-.02026655	.03390418
Operator 2	-.05096862	.090015324	-1.699	.128	-.12016052	.01822328
Operator 3	.01384396	.074443402	.558	.592	-.04337830	.07106622
**Yaw**						
Operator 1	.00938994	.035251689	1.865	.068	-.00073553	.01951541
Operator 2	-.05269350	.042265340	-3.740	**.006**	-.08518152	-.02020549
Operator 3	.00157049	.044599621	.106	.918	-.03271182	.03585279

Significant different was found for operator 2 in yaw.

### E. Reliability analysis on active and passive SP devices

**Fig 8:**
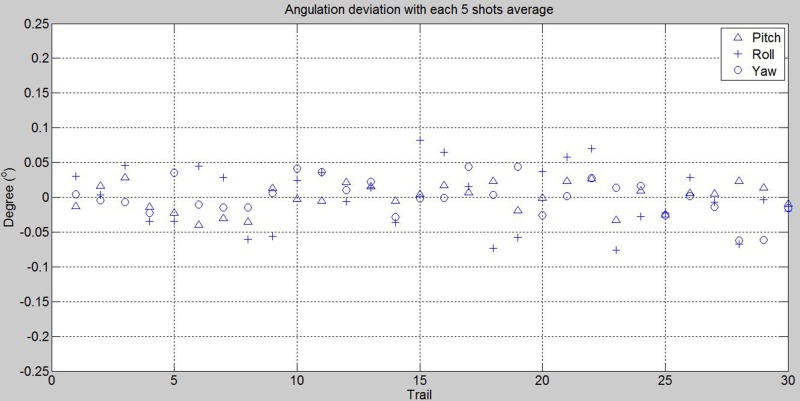
Reference board angulation deviations for using two front alignment lasers on 3dMD with 5 shots for each trail averaged.

**Fig 9:**
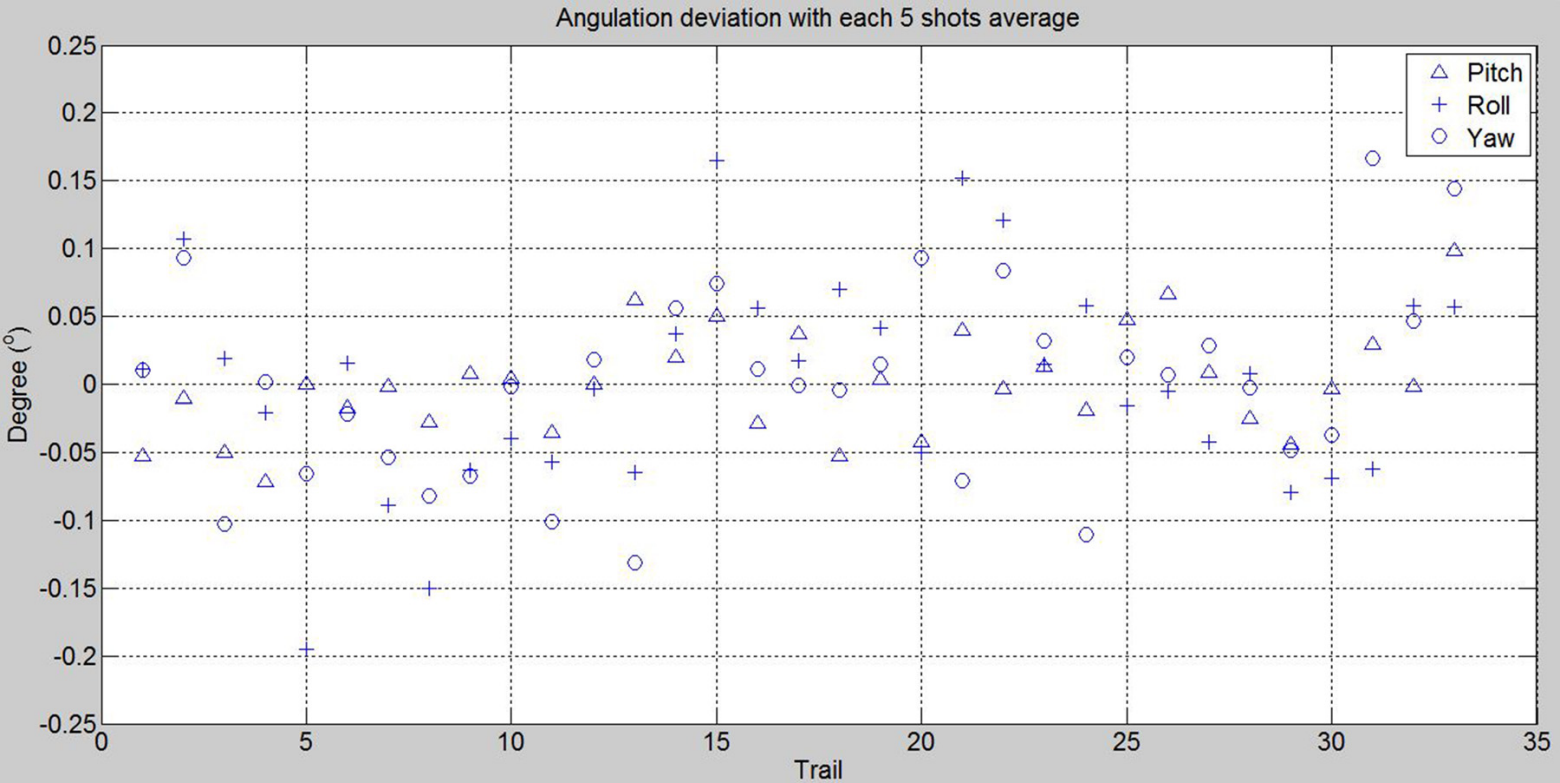
Reference board angulation deviations for using two front alignment lasers on DI3D with 5 shots for each trail averaged.

To measure the reliability for the active and passive SP devices, we performed (TCH, SWZ) reference board alignments with *M* = 5. That is, the detected axes were averaged with five shots in still for each alignment. In Figs 8 and 9, it shows the angulation deviations for *N* = 30 and 33 with averaged axes for using 3dMD and DI3D respectively. For 3dMD, the deviation ranges in pitch, roll and yaw were within ±0.039904°, ±0.081623°, ±0.062320°; and standard deviations 0.020234°, 0.045645° and 0.027211° respectively. For DI3D, the deviation ranges in pitch, roll and yaw were within ±0.098020°, ±0.195240°, ±0.166327°; and standard deviations 0.038850°, 0.079088° and 0.071816° respectively. The descriptive statistics of the experiments are shown in Table 4.

**Table 4:** Descriptive statistics of angulation deviations of reference board alignment for 3dMD (N = 30, M = 5) and DI3D (N = 33, M = 5) respectively.

**Angulation deviation for 3dMD**			**N = 30**	**M = 5**
	**SD/°**	**Median/°**	**Min/°**	**Max/°**
Pitch	0.020234	0.004771	-0.039904	0.028356
Roll	0.045645	-0.000016	-0.075797	0.081623
Yaw	0.027211	0.000187	-0.062320	0.044343
**Angulation deviation for DI3D**			**N = 33**	**M = 5**
	**SD/°**	**Median/°**	**Min/°**	**Max/°**
Pitch	0.038850	-0.001923	-0.071546	0.09802
Roll	0.079088	0.007572	-0.195240	0.164692
Yaw	0.071816	0.002099	-0.131241	0.166327

## Discussion

Traditional approaches to obtaining NHP involve capturing physical references and the patients’ images at the same time. Methods include placing markers on subjects’ face [19, 20]; holding orientation sensors with bite-jig [21, 22]; wearing eyeglasses mounted with inclinometers [23]; or hold spirit level equipped face bow [24] etc. The best accuracy achieved was ±1.1° [21, 22]. In using SP device, the proposed method captures physical references and patients’ images in separate scans [11]. This eliminates operator variability on applying references to patients and their adverse effects patients’ NHP. The major difference of the proposed approach to the traditional one is that, once the calibrations are done, the subsequent 3D scans with NHP are the same as normal. The patients can present their NHP in the most natural way.

In the proposed approach, two (or three) alignment lasers were used in the angulation adjustment steps. This enables simultaneous alignment in pitch, roll and yaw angles. Along with the adoption of 3D geared head and focusing adjuster, typically only 2.5 minutes is required for the whole setup. After aligning the reference board and 3D capture, the orientation axes are detected and saved. Subsequent patients’ facial models are then corrected automatically from the saved axes. There is no operator variability in the digital processing phase.

The patterns for reference axes detection were colored discs. They span almost 3/4 of the whole field of view with around 800–1500 vertex points for each orientation axes. The same alignment method and programs were used for the experiments with both of the SP devices. The only difference was that we added some textures on the colored discs pattern for DI3D. From the experiments, the reliability was improved by taking several shots of the board in still and averaging the detected axes. This can reduce the variability of orientations that is probably due to noise and data sampling. It is possible because the reference board can be kept still allowing multiple captures. On the other hands, other combinations of pattern are possible provided that the reference board is flat enough and the patterns are detectable by the SP device.

Integration of the orientation calibration to geometric camera calibration is also possible, provided that physical references are transferred and applied appropriately. In this paper, we demonstrated the idea of “orientation calibration” and suggested one solution using the 3D vertices and texture in the physical reference recording for NHP. It is compatible to the current SP devices (3dMD & DI3D) which uses different methods of geometric camera calibrations.

## Conclusion

In this paper, we presented a highly accurate and repeatable protocol for capturing physical references for recording NHP in stereo-photogrammetry. The suggested additional orientation calibration step is performed after the device geometric camera calibration. No devices or markings are needed to be placed on patients. It can be applied in most of the commercially available SP devices. Orientation-calibrated stereo-photogrammetry is the most accurate method (angulation deviation within ±0.1°) reported for complete NHP recording with insignificant clinical error.

## Supporting Information

S1 File ZIP archive of The reference boards in digital form (Adobe psd and pdf). (ZIP)

S2 File ZIP archive of the compiled simulation programs and test data. (ZIP)

S3 File ZIP archive of simulation source code (Matlab). (ZIP)

S4 File ZIP archive of the experiment data. (ZIP)
