# Supplementary figures and images for: Automatic Detection and Reproduction of Natural Head Position in Stereo-Photogrammetry
Source: PLoS One. 2015 Jun 30;10(6):e0130877. doi: 10.1371/journal.pone.0130877 (PMC4488321; doi:10.1371/journal.pone.0130877)

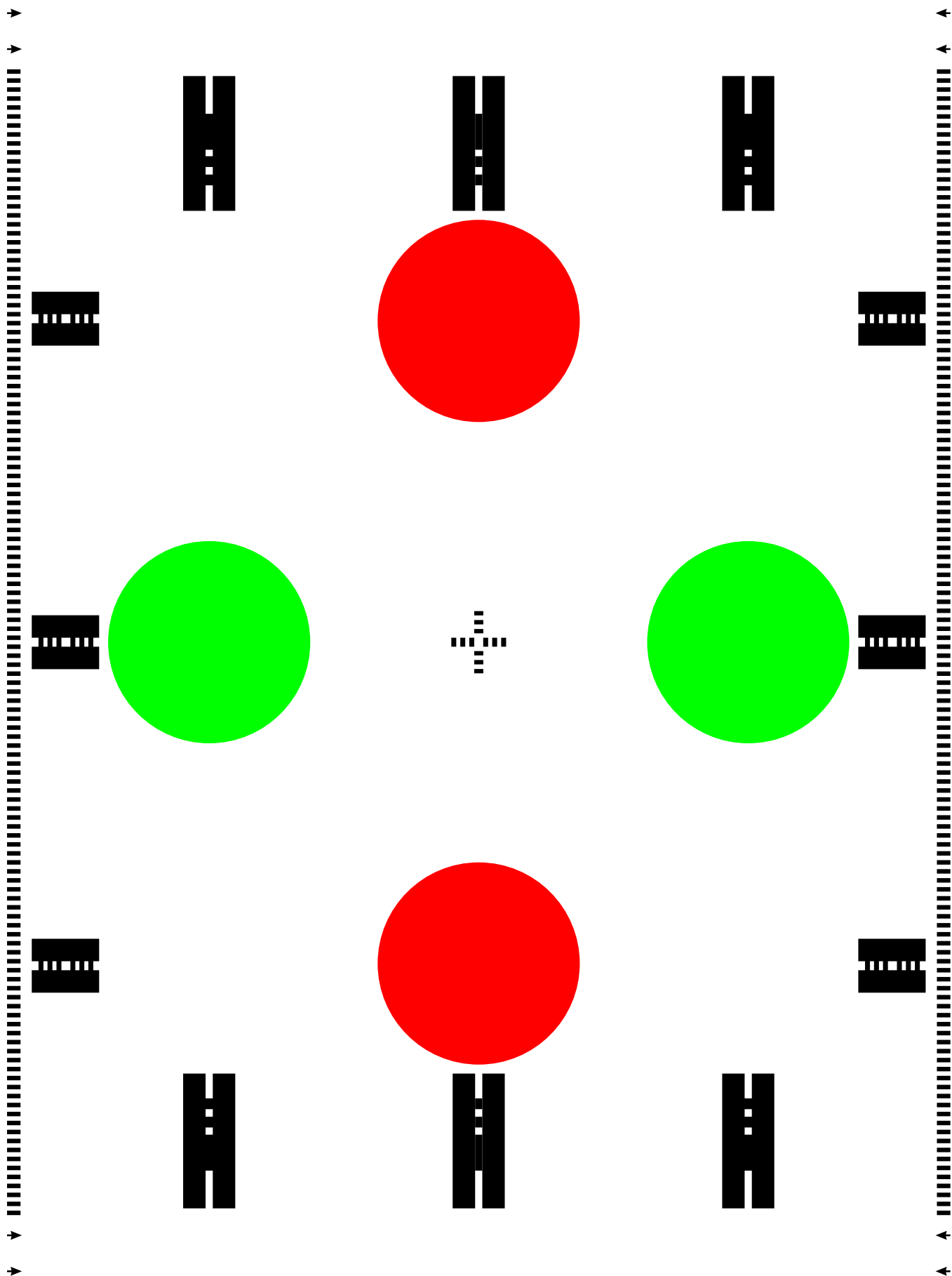

Supplement: S1 File — (ZIP) [file pone.0130877.s001.zip › ref-board/nhp_Disc-VRed-HGreen-9Cross.pdf]

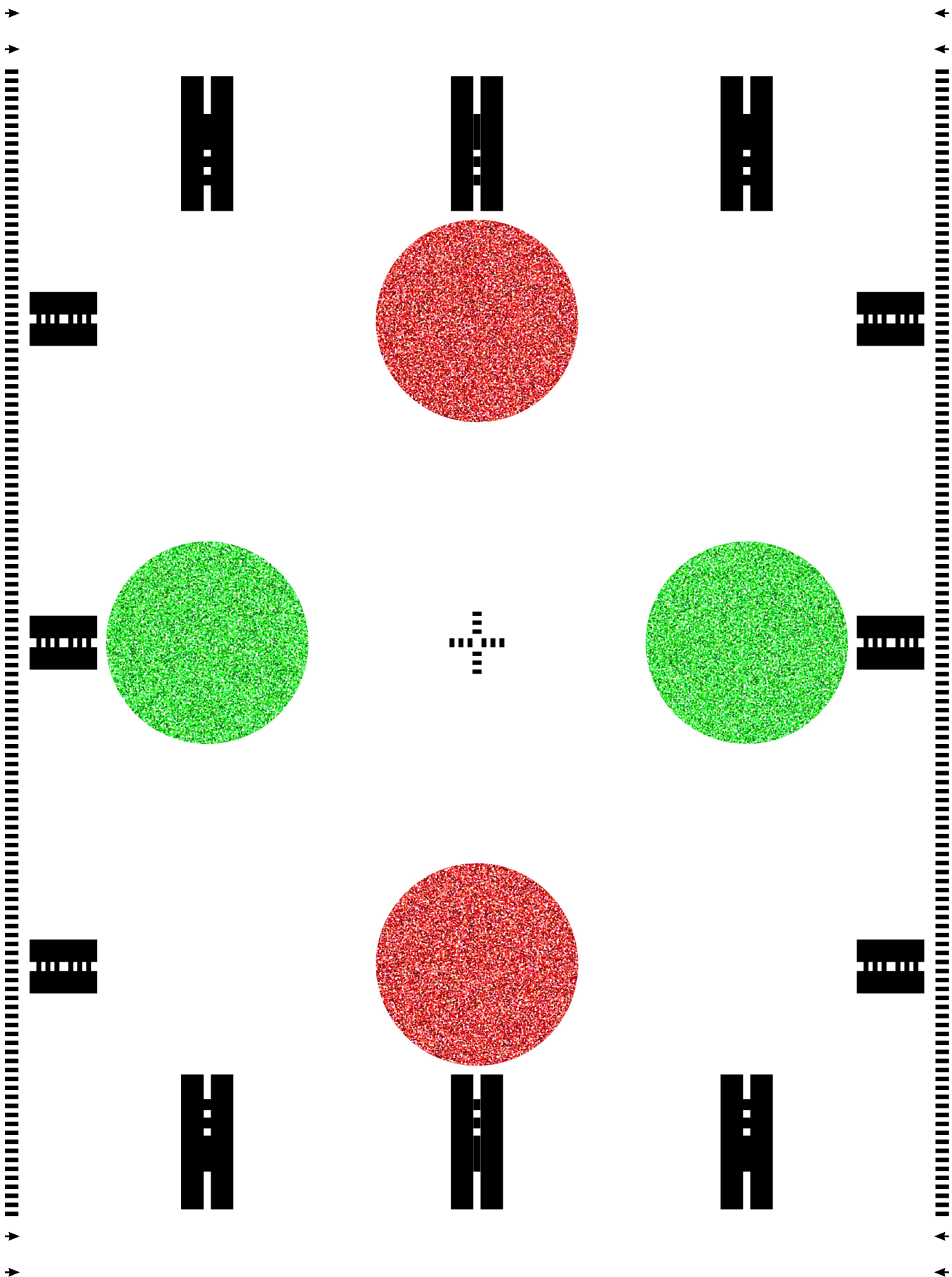

Supplement: S1 File — (ZIP) [file pone.0130877.s001.zip › ref-board/nhp_Disc-VRed-HGreen-9Cross_pt5.pdf]

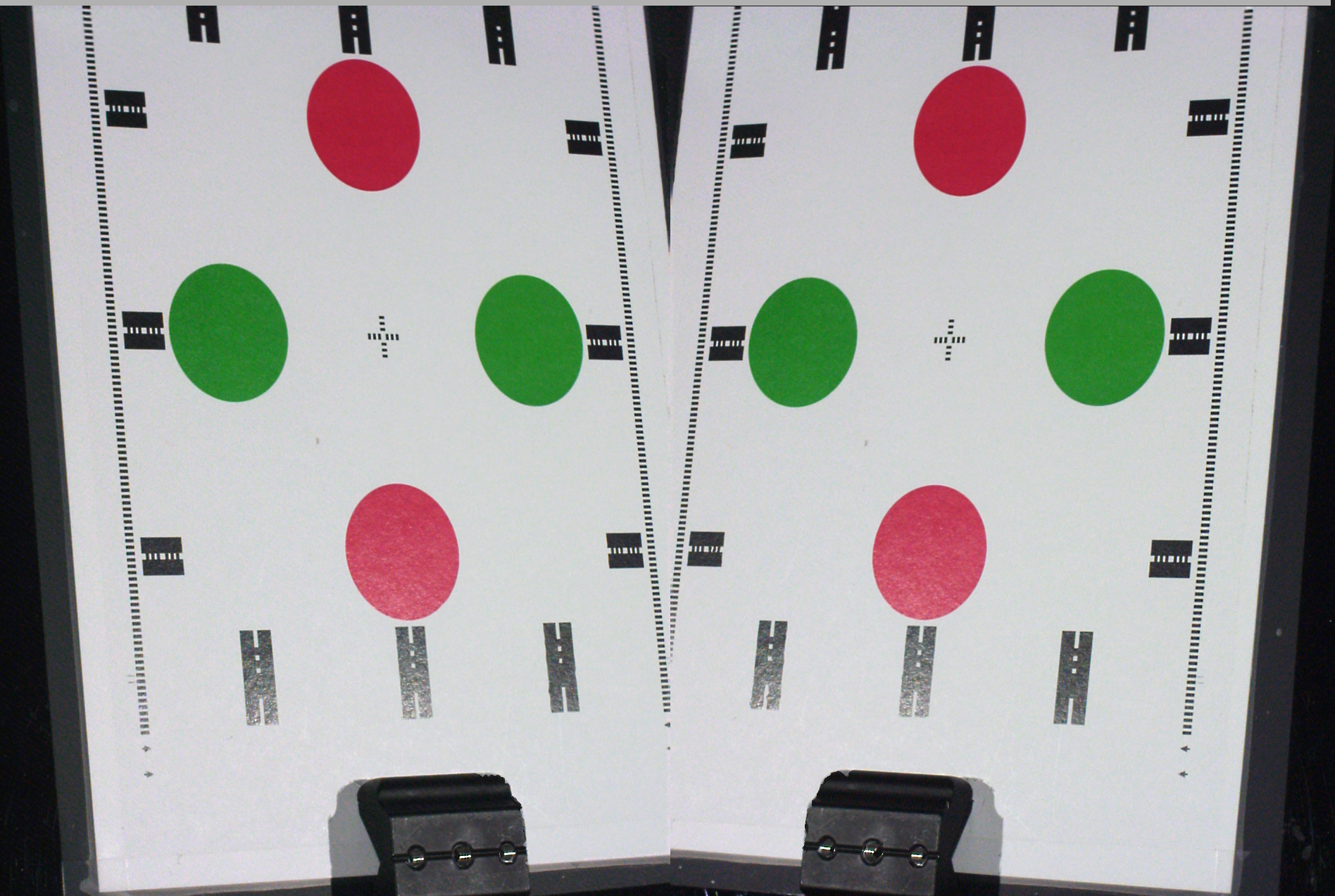

Supplement: S2 File — (ZIP) [file pone.0130877.s002.zip › autoSPNHP/3dmd/140918171945.bmp]

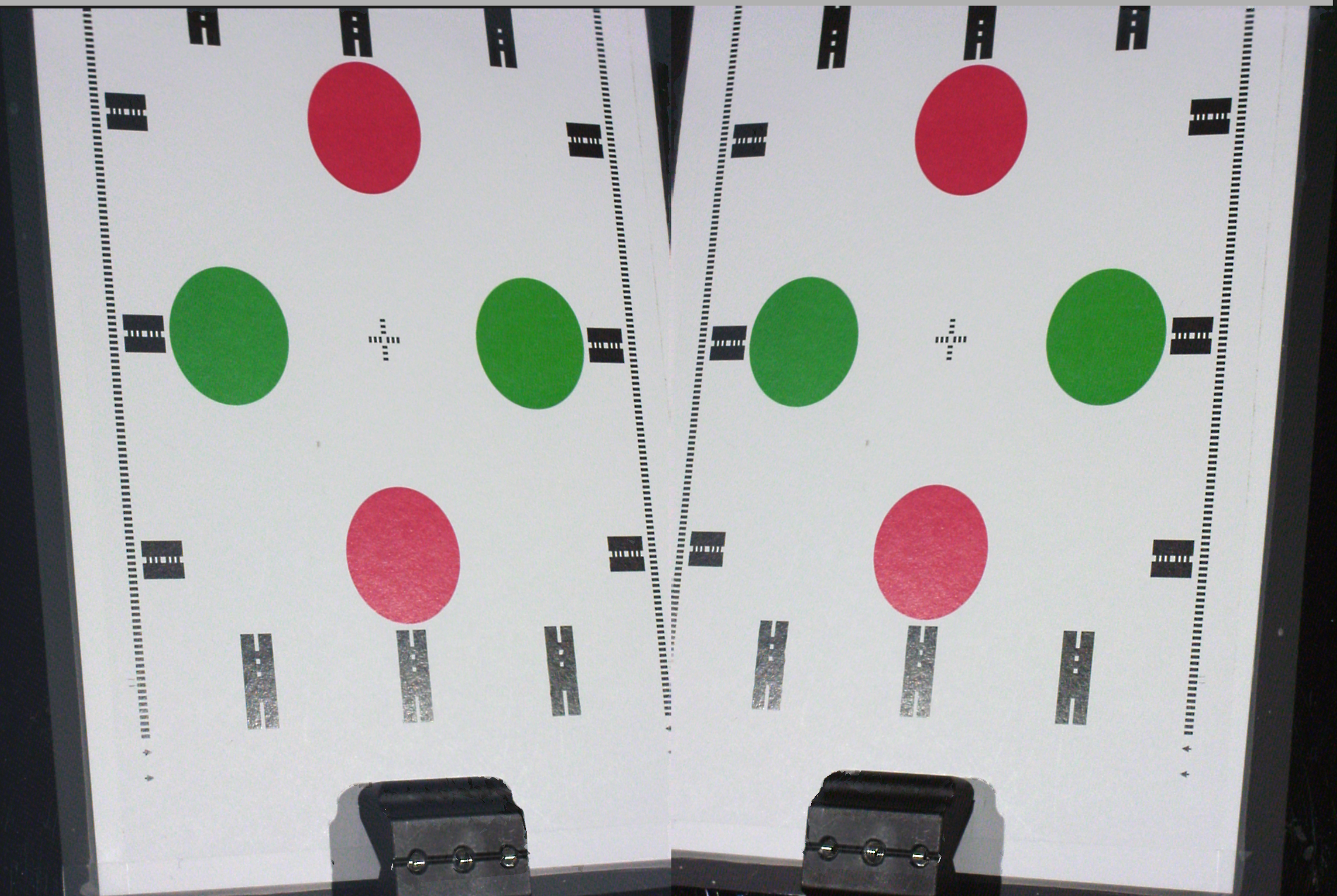

Supplement: S2 File — (ZIP) [file pone.0130877.s002.zip › autoSPNHP/3dmd/140918172023.bmp]

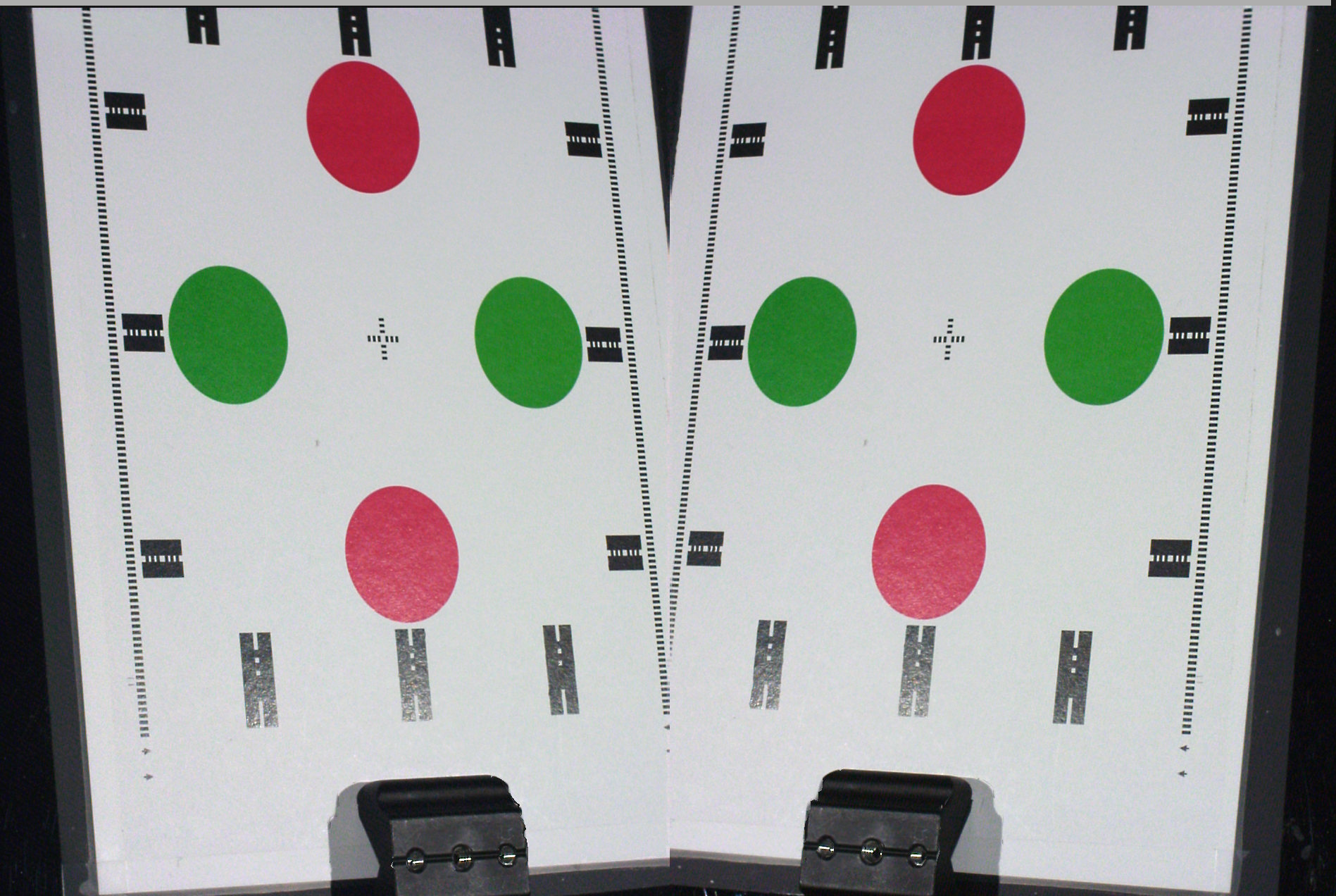

Supplement: S2 File — (ZIP) [file pone.0130877.s002.zip › autoSPNHP/3dmd/140918172053.bmp]

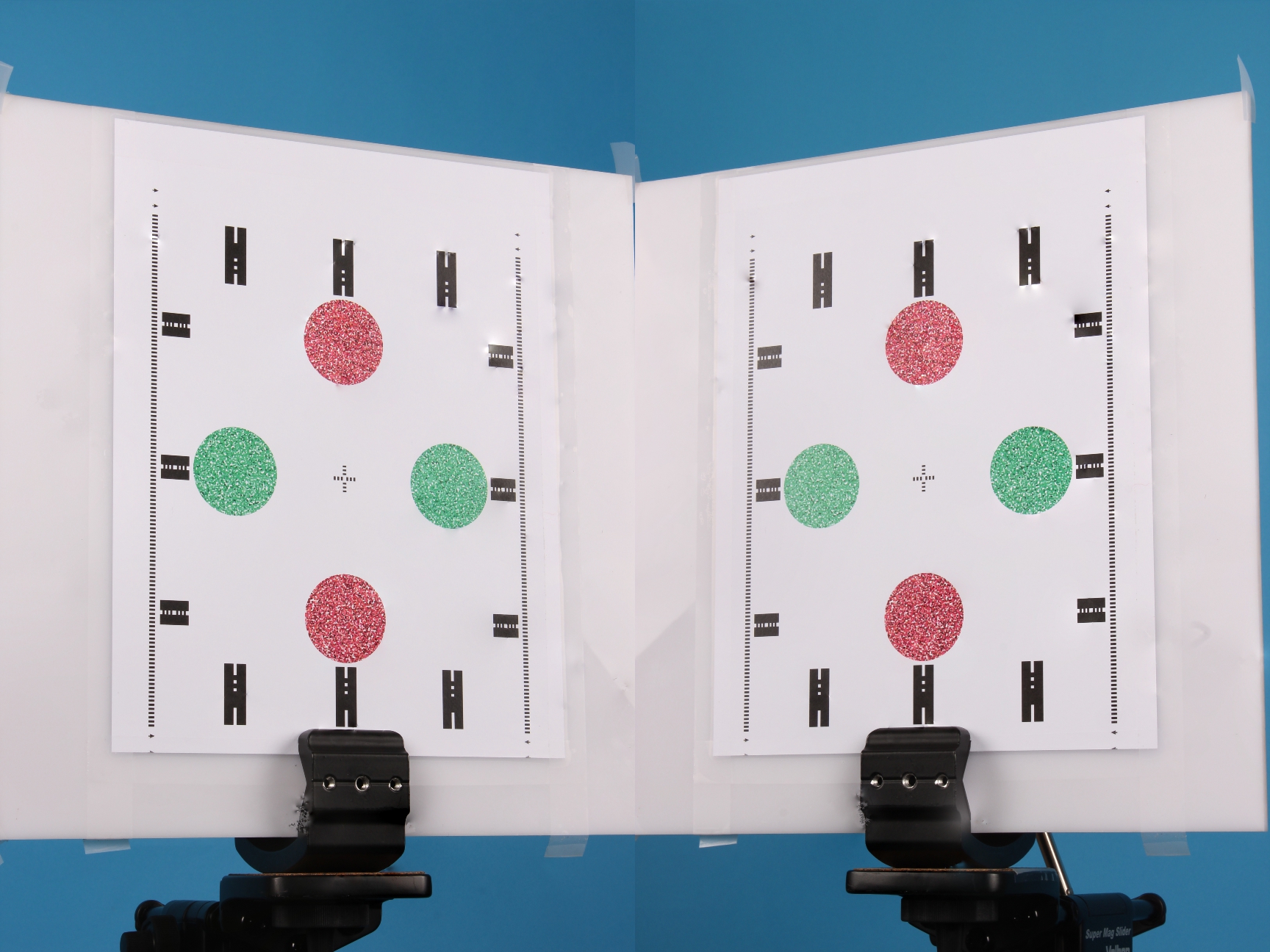

Supplement: S2 File — (ZIP) [file pone.0130877.s002.zip › autoSPNHP/di3d/board_15-10_001.jpg]

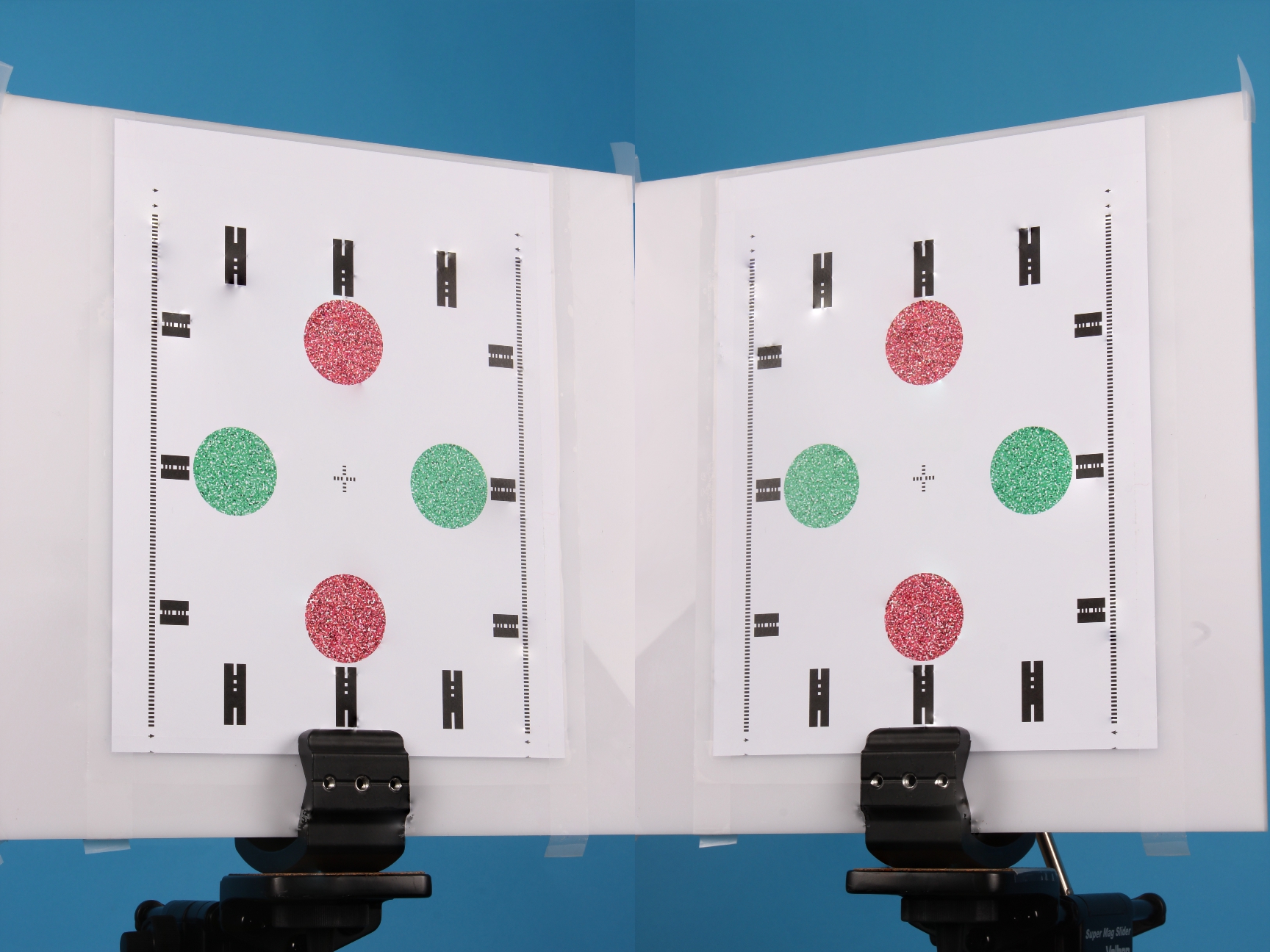

Supplement: S2 File — (ZIP) [file pone.0130877.s002.zip › autoSPNHP/di3d/board_15-10_002.jpg]

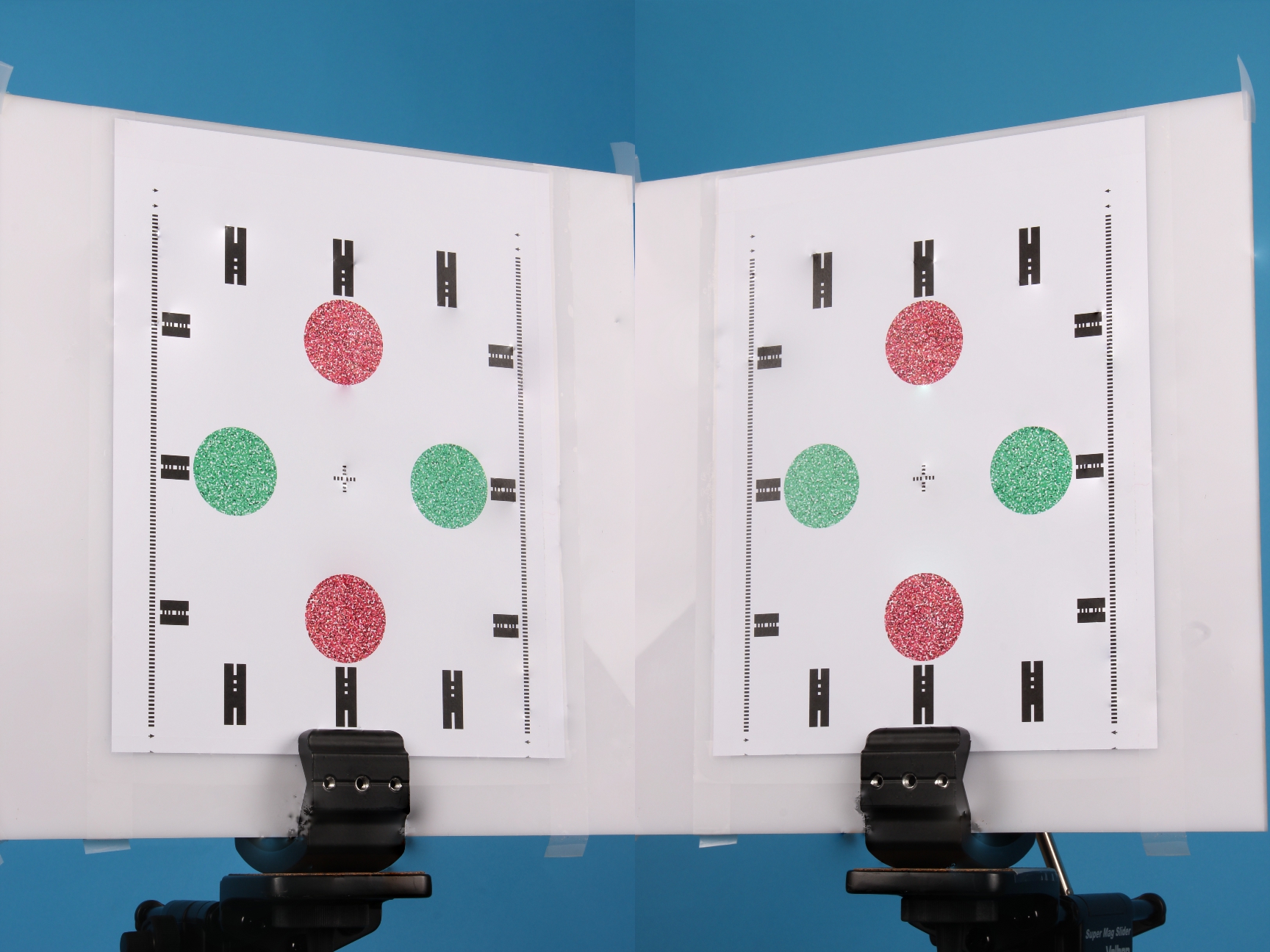

Supplement: S2 File — (ZIP) [file pone.0130877.s002.zip › autoSPNHP/di3d/board_15-10_003.jpg]

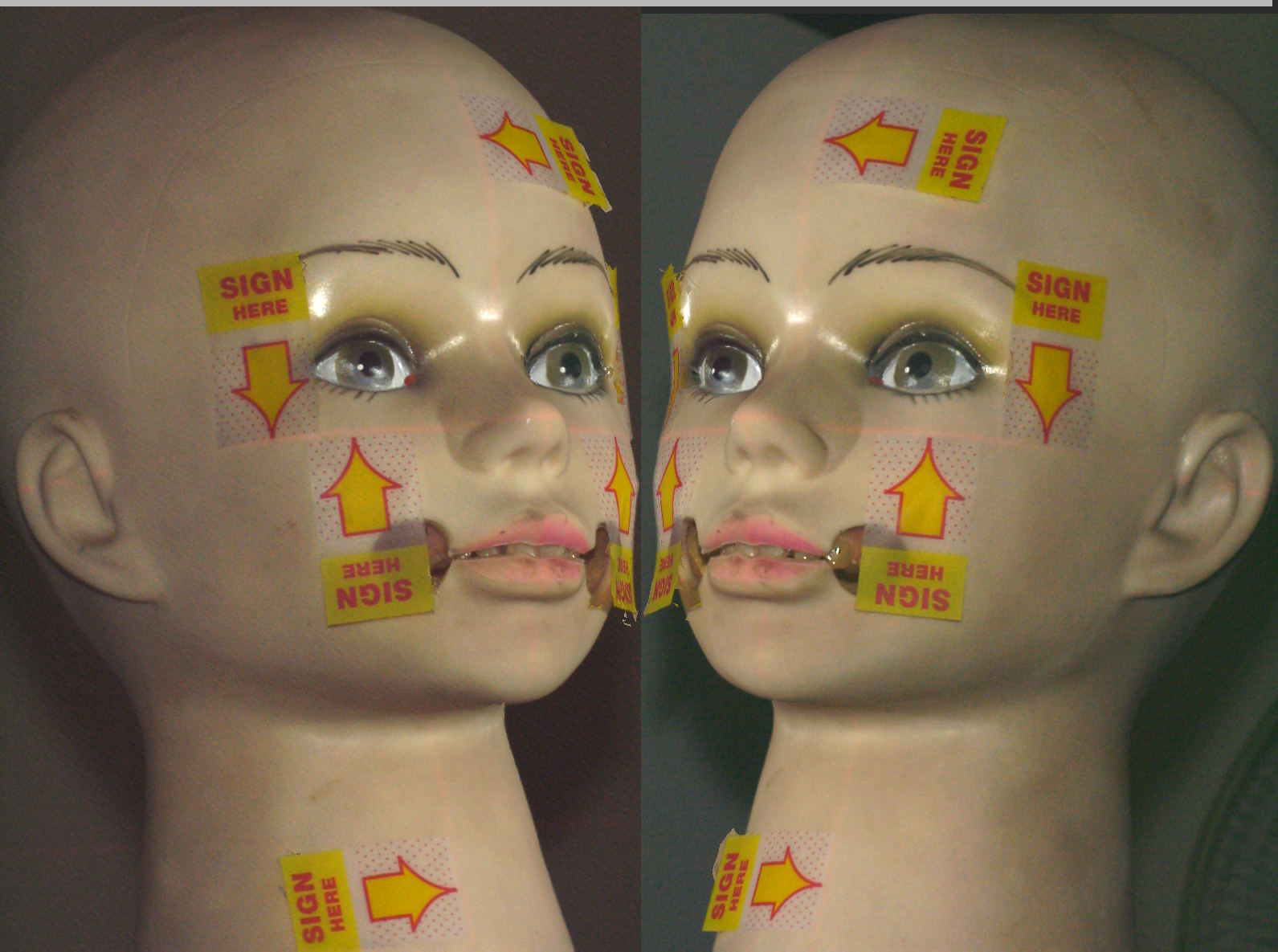

Supplement: S2 File — (ZIP) [file pone.0130877.s002.zip › autoSPNHP/plastichead/140925161808.bmp]
